# Glioblastoma-Derived Small Extracellular Vesicles: Nanoparticles for Glioma Treatment

**DOI:** 10.3390/ijms24065910

**Published:** 2023-03-21

**Authors:** Salomé Araujo-Abad, Antonio Manresa-Manresa, Enrique Rodríguez-Cañas, María Fuentes-Baile, Pilar García-Morales, Ricardo Mallavia, Miguel Saceda, Camino de Juan Romero

**Affiliations:** 1 Instituto de Investigación, Desarrollo e Innovación en Biotecnología Sanitaria de Elche (IDiBE), Universidad Miguel Hernández de Elche, 03202 Alicante, Spain; 2 Centro de Biotecnología, Universidad Nacional de Loja, Loja 110111, Ecuador; 3 Fundación para el Fomento de la Investigación Sanitaria y Biomédica de la Comunidad Valenciana (FISABIO), Hospital General Universitario de Elche, Unidad de Investigación, 03203 Alicante, Spain

**Keywords:** glioblastoma, small EVs, nanocarriers, temozolomide, EPZ015666, FESEM

## Abstract

Glioblastoma (GBM), characterized by fast growth and invasion into adjacent tissue, is the most aggressive cancer of brain origin. Current protocols, which include cytotoxic chemotherapeutic agents, effectively treat localized disease; however, these aggressive therapies present side effects due to the high doses administered. Therefore, more efficient ways of drug delivery have been studied to reduce the therapeutic exposure of the patients. We have isolated and fully characterized small extracellular vesicles (EVs) from seven patient-derived GBM cell lines. After loading them with two different drugs, Temozolomide (TMZ) and EPZ015666, we observed a reduction in the total amount of drugs needed to trigger an effect on tumor cells. Moreover, we observed that GBM-derived small EVs, although with lower target specificity, can induce an effect on pancreatic cancer cell death. These results suggest that GBM-derived small EVs represent a promising drug delivery tool for further preclinical studies and potentially for the clinical development of GBM treatments.

## 1. Introduction

Glioblastoma (GBM) is the most aggressive primary malignant tumor of the central nervous system. According to the World Health Organization (WHO), it corresponds to IDH-wildtype grade 4 and is mainly present in adults, with a median survival up to 15 months [1,2]. This short survival rate can be attributed to treatment limitations. The standard of care for GBM patients is the combination of radiotherapy and chemotherapy with temozolomide (TMZ) (3,4-dihydro-3-methyl-4-oxoimidazo-[5,1-d]-astetrazine-8-carboxamide) after tumor resection [3,4]. TMZ is a DNA alkylating agent that acts over the methylation of guanine and adenine bases, breaking double-stranded DNA and causing cell cycle arrest and cell death by apoptosis [5]. Despite its mighty effect on GBM, there are some disadvantages associated with TMZ treatment, such as its short half-life, the requirement of high doses to achieve therapeutic levels, and a great number of side effects [6], among them, headache, fatigue, loss of appetite, opportunistic infections, thrombocytopenia, moderate to severe lymphopenia, and abnormally low levels of white blood cells [7,8,9]. More importantly, only 20% of TMZ, with respect to a systemic dose, reaches the brain [10].

Recently, arginine methyltransferase-5 (PRMT5) has been described as a potential target for cancer treatment. PRMT5 modulates the biological function of target proteins, catalyzing the transference of two methyl groups to arginine residues, and is essential to maintain homeostasis in both normal and malignant cells [11,12]. This enzyme is involved in tumorigenesis, and it has been found overexpressed in a variety of cancers, including melanoma, multiple myeloma, lung, gastric, prostate, ovarian, colorectal cancers, and GBM [13]. Moreover, the overexpression of this protein has been correlated with poor patient prognosis [12]. EPZ015666 is a small molecule that competes with the substrate-binding pocket of the PRMT5 peptide to deny its interaction and posterior methylation; therefore, it has been considered a PRMT5 inhibitor [14].

The delivery of therapeutic agents to brain tumors is particularly challenging, with no improvement in outcomes observed over the last 20 years, since the Stupp protocol was shown to extend the life span of patients for just several months [3]. This is in contrast to the cancer of other organs that have undergone huge therapeutic improvements over the last decade. Among the new therapies, a novel alternative is the use of exosomes as drug carriers that can contribute to increasing the efficiency of treatments and enhancing brain targeting. Exosomes are small extracellular vesicles (EVs) that were first identified as “trash bags”, a system in which waste cellular products were disposed of [15]. However, diverse studies have confirmed that small EVs play a major role in cell–cell communication, both in normal conditions and in pathologies, such as cancer [16]. Small EVs can be used as nanocarriers with some advantages, such as specific tropism for cell origin, non-cytotoxic effect, biocompatibility, facility of transporting hydrophilic or hydrophobic biomolecules, and facility to cross the blood–brain barrier (BBB) [16,17,18,19]. Small EVs have a diameter of 30–150 nm and a lipid bilayer membrane with the same characteristics as the donor cell, and their cargo is made up of proteins, messenger RNA (mRNA), microRNA (miRNA), long non-coding RNA (lncRNA), DNA fragments, and metabolites from the donor cell [16,20,21,22]. Small EVs can be characterized by the presence of proteins, such as Alix, TSG101, RAB, small GTPases, flotillins, annexins, and CD9, CD63, and CD81. The presence or absence of these is closely related to the cell from which the small EVs originated [23,24,25].

In the present study, we focused on small EVs released from previously isolated, patient-derived GBM cells [26]. We described an efficient method of small-EV purification from those GBM cell lines that we used, and the purity of the small EVs population in our samples has been ensured by performing several characterization methods. We have used those small EVs to overcome the present challenges of drug delivery in GBM and to improve the delivery system by testing two procedures in order to load them with chemotherapeutic drugs, TMZ and EPZ015666. We demonstrate that small EVs loaded with each drug inhibit the proliferation of GBM more efficiently than the drug alone. Therefore, GBM-derived small EVs are not only valuable as a prognostic marker for GBM, but also as delivery systems for the development of new therapeutic strategies.

## 2. Results

### 2.1. Sensitivity and/or Resistance Profile to Treatments of Patient-Derived GBM Cell Lines

**Figure 1:**
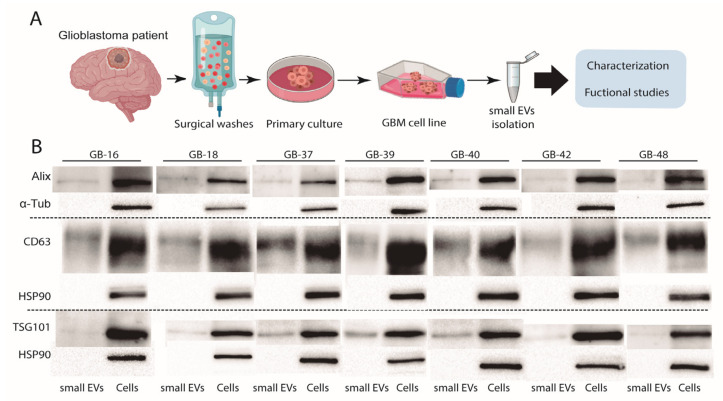
**Small EVs isolation and biochemical characterization.** (**A**) Scheme of the GBM cell line obtention, followed by small EVs isolation and functional studies. (**B**) Expression by WB of Alix, CD63, and TSG 101 in isolated small EVs and corresponding cell protein extracts. α-Tub and HSP-90 were used as control. Of note, HSP-90 in GBM is not a biomarker and has been used as a marker of small EVs isolation (negative control). GBM, glioblastoma; EVs, extracellular vesicles.

The GBM cell lines used in this work were obtained by our research group, as described previously, by surgical washes of resection surgery from patients older than 18 years diagnosed with GBM, and all of them IDH wt. They were denominated “Hospital General Universitario de Elche” (HGUE), Glioblastoma (GB), and the number of the patient (Figure 1A) [26]. Thus, seven cell lines were established: HGUE-GB-16, HGUE-GB-18, HGUE-GB-37, HGUE-GB-39, HGUE-GB-40, HGUE-GB-42, and HGUE-GB-48 (indicated in the Figures as GB-patient number).

We performed a proliferation assay that showed an important decrease in proliferation as a response to TMZ treatment by all GBM cell lines (Figure S1). TMZ was the most effective treatment, together with EPZ015666, which also showed a significant response in the GBM cell lines (Figure S2).

Those were the only treatments to which no GBM cell line showed resistance. Regarding the GBM cell lines, the most sensitive to all treatments was HGUE-GB-42, whereas HGUE-GB-39 was the most resistant, together with HGUE-GB-16. The different sensitivity/resistance profiles of the cell lines derived from several patients suffering from the same type of tumor highlights the importance of better analysis and understanding of the tumor features in order to find common properties and, therefore, overcome GBM therapy limitations (Table 1).

### 2.2. Small EVs Isolation and Biochemical Characterization of Small EVs from Patient-Derived GBM Cell Lines

There are many procedures for small EVs isolation based on different biophysical and biochemical properties of EVs, such as size, mass density, shape, charge, or antigen exposure [23,27].

**Figure 2:**
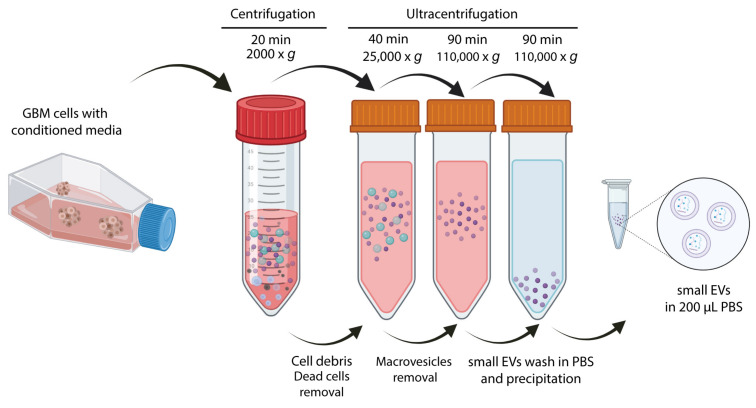
**Scheme representing GBM-derived small EVs isolation.** Sequential steps of centrifuge and ultracentrifuge were performed, as indicated on top of the arrows. GBM, glioblastoma; EVs, extracellular vesicles.

In order to isolate small EVs from GBM cell lines, the protocol was adapted according to the small EVs’ origin and experiment type. In this study, we used the ultracentrifugation approach for small EVs isolation (Figure 2).

Small EVs can mirror their cellular origin and, therefore, can provide us with critical information associated with the physiological state of their parental cell [28].

The standardization of small EVs characterization is an essential step for reliable and reproducible results from assays and other downstream applications. In order to characterize our samples, we used Western Blot (WB) as an analytical technique, with antibodies against the main proteins present in small EVs. Our results showed the presence of typical small EVs proteins, such as Alix (Invitrogen), TSG101 (Invitrogen), and CD63 (Invitrogen), while α-Tubulin (Invitrogen) and HSP-90 (CUSABIO) were only found in cell lysates (Figure 1B), in contrast to other results obtained with small EVs purified from breast or prostate cancer cells [29,30]. Furthermore, the absence of tubulin was reported as a characteristic of small EVs purity, indicating the absence of cell contaminants in the sample [31]. Altogether, our results established the purity of the small EVs samples. Moreover, we could identify the expression of HSP-90 in the GBM cell lines, but not in the GBM-derived small EVs samples, which differs from small EVs of different origin.

### 2.3. Morphological Characterization of GBM-Derived Small EVs

**Figure 3:**
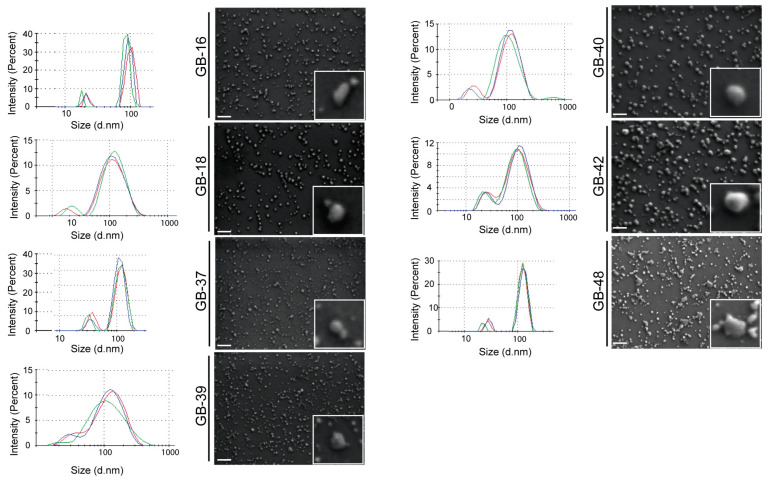
**Size characterization of small EVs particles.** Size and morphology of the seven GBM cell lines’ small EVs were visualized by using Dynamic Light Scattering (DLS) and Field Emission Scanning Electron Microscopy (FESEM). White boxes show an optical magnification of a representative small EV. Scale bars 200 nm. GBM, glioblastoma; EVs, extracellular vesicles.

Characterization of isolated GBM-derived small EVs was completed by Dynamic light scattering (DLS) (Figure 3) and showed that the isolated particles from the seven patients’ samples were consistently within the expected size range for small EVs (Figure 3). Analysis revealed different diameter means according to each group of cell-line-derived small EVs: HGUE-GB-16 (93 nm ± 7), HGUE-GB-18 (126 nm ± 3), HGUE-GB-37 (117 nm ± 5), HGUE-GB-39 (129 nm ± 1), HGUE-GB-40 (118 nm ± 6), HGUE-GB-42 (110 nm ± 6), and HGUE-GB-48 (126 nm ± 3).

We further examined the size and morphology of GBM-derived small EVs by performing field emission scanning electron microscopy (FESEM) analyses (Figure 3). FESEM images indicated the presence of a homogeneous population of small EVs, with a mean diameter between 100 and 150 nm. Small EVs appeared almost exclusively as round or cup-shaped vesicles. In some cases, we could identify multiple small EVs clusters of about 200 nm in diameter.

To rule out that the structures visualized by FESEM could be an artifact of the isolation or fixation process, we performed an exhaustive analysis of the samples. Energy dispersive X-ray (EDX) analysis was performed with the X-ray detector in a FESEM microscope on a HGUE-GB-48-derived small EVs sample (Figure S3). As expected, the studied sample was a mixture of biological samples and salts. The spectrum of the rounded, small EVs-like areas confirmed their biological origin. Taken together, DLS and FESEM imaging confirmed the purity of the GBM-derived small EVs in our preparations and are in line with similar studies uncovering small EVs characteristics derived from cells of diverse origins [32]. Moreover, for the first time, EDX analysis was performed to allow the discrimination of small EVs structures from salts by establishing the biological nature of the samples (Figure S3).

### 2.4. Effect of GBM-Derived Small EVs Directly Loaded on Glioma Cells

The current standard-of-care treatment for GBM relies on a multidisciplinary approach combining surgery, chemotherapy, and radiation therapy. TMZ has been used as a first-line treatment for GBM. On the other hand, chemoresistance has become the main barrier to treatment success, and therefore, other drugs have been developed to treat GBM patients [3]. Our data showed that patient-derived GBM cell lines were mostly sensitive to TMZ and EPZ015666; thus, we decided to test both drugs. EPZ015666 is an inhibitor of PRMT5 that has been shown to regulate the splicing of detained introns in proliferation-associated genes in GBM [33]. PRMT5 has a nuclear localization, and its expression correlates with poor survival in several types of cancers. This makes PRMT5 a good candidate for cancer therapy, and consequently, many compounds have been screened to find the required specificity and efficacy for GBM treatment [34]. One of them is EPZ015666, which has been broadly used in several other types of cancer [14,35]. In this study, we tested the effect of EPZ015666 on GBM cell survival.

In order to test whether or not GBM-derived small EVs can be used as a drug delivery system for cancer treatment, we first loaded them by two incubation methods [36]. The first method has been broadly used to load small EVs and consists in the treatment of the cells for at least 48 h with high doses of a certain drug, followed by small EVs isolation from the medium [19]. From now on, we will call this method “indirect incubation” [37]. In the second one, called “direct incubation”, small EVs are first isolated from the cell line and then incubated in a medium containing a low dosage of the drug [38].

To test the loading efficiency of small EVs, the small EVs were loaded by direct and indirect incubation, and the amount of TMZ was measured by HPLC (Figure S4). Small EVs isolated from the GBM cell line HGUE-GB-39 were isolated (Figure 2), and the drug was encapsulated by direct incubation in 5 mM TMZ. Our HPLC measurements showed higher amounts of TMZ being loaded into the small EVs by direct incubation; therefore, we decided to continue the experiments with this loading method (Figure S4).

**Figure 4:**
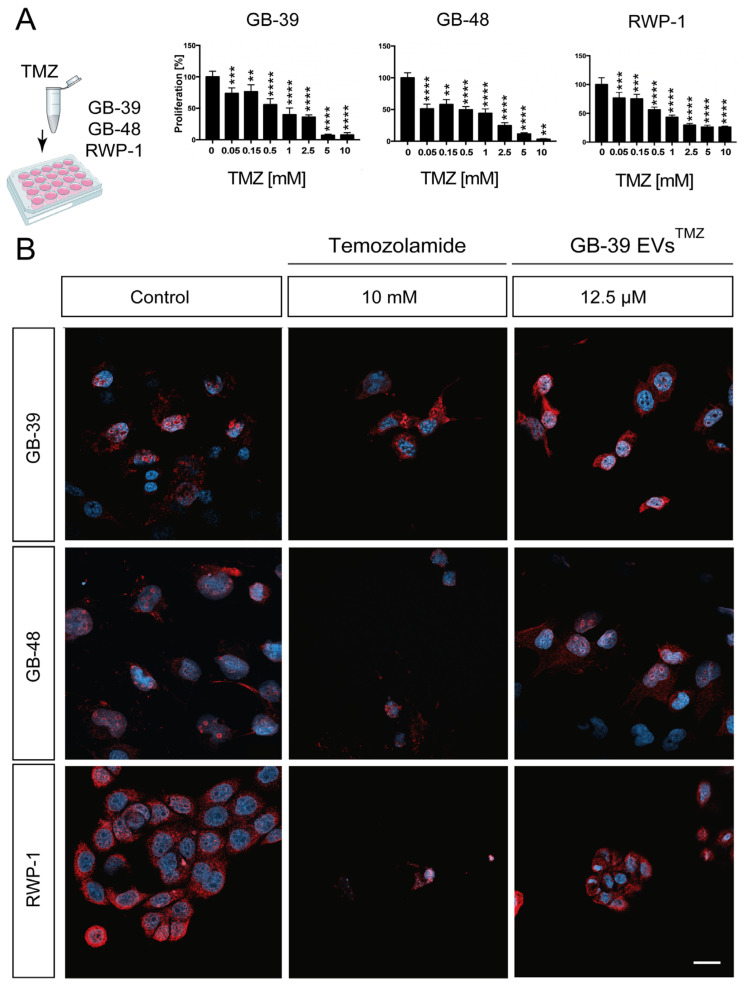
**Cell proliferation effect of TMZ in cancer cell lines.** (**A**) Cancer cells were treated with increasing concentrations of TMZ, and their proliferation was measured. (**B**) Confocal images showing the expression of a proliferative marker and the decrease in cell density due to the TMZ and GB-39 EVs^TMZ^ effect. Asterisks indicate the statistical significance of the results (** *p* < 0.01, *** *p* < 0.001, **** *p* < 0.0001). Scale bar B, 20 μm. TMZ, Temozolomide; EVs, extracellular vesicles.

**Figure 5:**
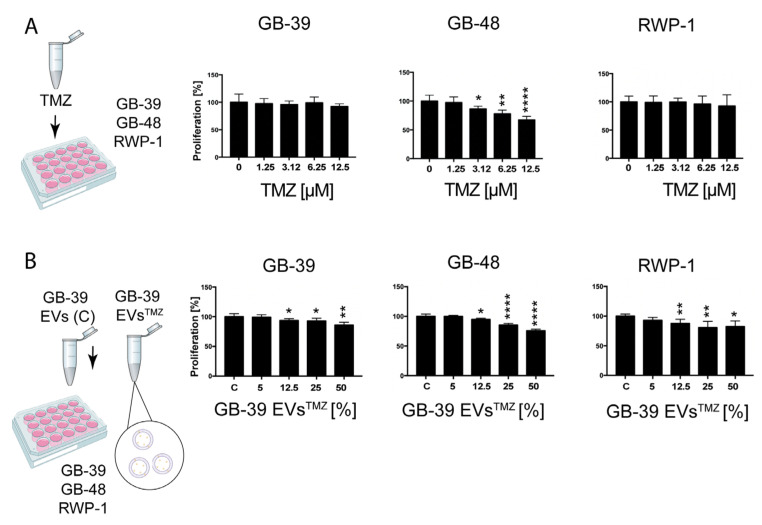
**Cell proliferation effect at low concentrations of TMZ in cancer cell lines.** (**A**) Cancer cells were treated with low concentrations of TMZ, similar to the calculated concentrations present in GB-39 EVs^TMZ^, and their proliferation was measured. (**B**) GB-39 EVs^TMZ^ were loaded directly with a 5 mM concentration of TMZ. Small EVs alone (Control, C) or serial dilutions of the loaded small EVs were applied to different cancer cell lines, and their effect on proliferation was measured. Asterisks indicate the statistical significance of the results (* *p* < 0.05, ** *p* < 0.01, **** *p* < 0.0001). TMZ, Temozolomide; EVs, extracellular vesicles; C, control.

**Figure 6:**
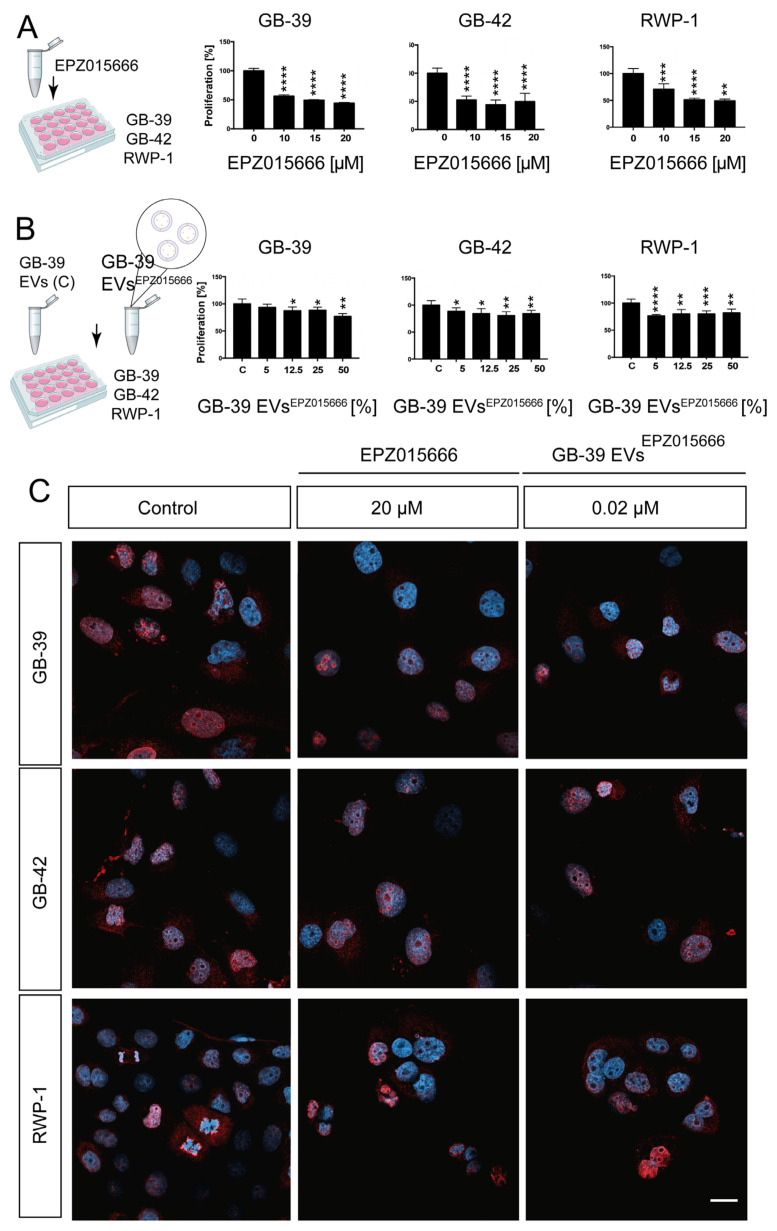
**Cell proliferation effect of EPZ015666 in cancer cell lines.** (**A**) Cancer cells were treated with increasing concentrations of EPZ015666, and their proliferation was measured. (**B**) Small EVs of GB-39 were loaded directly with a 15 μM concentration of EPZ015666. Small EVs alone or serial dilutions of the loaded small EVs were applied to different cancer cell lines, and their effect on proliferation was measured. (**C**) Confocal images showing the expression of a proliferative marker and the decrease in cell density due to the EPZ015666 and GB-39 EVs^EPZ015666^ effect. Asterisks indicate the statistical significance of the results (* *p* < 0.05, ** *p* < 0.01, *** *p* < 0.001, **** *p* < 0.0001). Scale bar C, 20 μm. EVs, extracellular vesicles.

Small EVs have been proposed to tend to fuse preferentially with their parent cancer cells [17]. To test this idea, we included in our experiment not only the parental cell HGUE-GB-39, but also another GBM cell line and the unrelated pancreatic cancer cell line RWP-1. The second GBM cell line was chosen based on its sensitivity/resistance profile against each drug (Figure S1). The HGUE-GB-48 GBM cell line was used for TMZ treatment, whereas EPZ015666 was applied to HGUE-GB-42, since they were the most sensitive lines against those drugs, according to our data (Table 1). The above-mentioned three cancer cell lines were treated with small EVs derived from the GB-39 GBM cell line that was loaded by the direct incubation method, and the effect on cell proliferation was measured. When applying the loaded small EVs in the same or in another GBM cell line, direct incubation showed a significant effect, as indicated by the decrease in cell proliferation, especially at higher concentrations (Figure 4, Figures S5 and S6). Surprisingly, RWP-1 also responded to drug-loaded small EVs. This may be explained by the fact that RWP-1 is very sensitive to both of the tested drugs, even at low doses, as shown in the direct exposure to both drugs (Figure 4, Figure 5 and Figure 6, Figures S5, S6, S9, and S10).

### 2.5. TMZ-Loaded GBM-Derived Small EVs Barely Affect Cancer Cells

The effect of TMZ treatment on proliferation was assessed using an increasing concentration of the drug. We used three cancer cell lines, two of GBM and one of pancreatic cancer, to evaluate cell tropism later. A range of concentrations between 0.05 mM and 10 mM was evaluated. Our analyses revealed that treatment of the GBM cell lines HGUE-GB-39 and HGUE-GB-48 with TMZ resulted in more than 90% decreased proliferation, together with an IC_50_ of 1.088 ± 0.082 and 0.2264 ± 0.195 mM, respectively (Figure 4, Figures S5 and S6). Interestingly, the pancreatic cancer cell line RWP-1 was also affected by the TMZ administration, with only 20% of proliferating cells at 10 mM concentrations and an IC_50_ of 0.7301 ± 0.066 mM (Figure 4, Figures S5 and S6).

We then tested the second loading method by directly incubating GB-39-derived small EVs in 5 mM TMZ (GB-39 EVs^TMZ^). It has been described that this kind of incubation results in a very low encapsulation rate of the administered drug dose within the small EVs [36]. In order to know the precise amount of drug contained in the small EVs, we performed HPLC analysis, followed by drug identification with a mass spectrometer (Figure S4). Our quantifications showed that GB-39 EVs^TMZ^ contained 25 μM of the drug. Isolated GB-39 EVs^TMZ^ were applied to the cells in serial dilutions ranging from 50% to 5% of the original stock. Since the amount of drug applied to the cultures through the GB-39 EVs^TMZ^ corresponds to a range of 12.5 μM to 1.25 μM, we tested the direct exposure of the GBM cell lines to TMZ at these low concentrations to compare both effects (Figure 5, Figure S5 and S6).

The maximum concentration applied was 12.5 μM of TMZ, corresponding to 50% GB-39 EVs^TMZ^, and the minimum was 1.25 μM at 5% of TMZ. GB-39 EVs^TMZ^ treatment resulted in a very subtle reduction in cancer cell proliferation. Interestingly, the total amount of TMZ used for this treatment was up to 1000 times less than the direct application of TMZ in cells (Figure 5B) already starting at a 6 μM concentration.

Moreover, our HPLC data indicated that the amount of drug loaded into isolated GB-39 EVs^TMZ^ (Figure S4) was 200 times less than the concentration they were exposed to while being incubated by the direct method (Figure S4). On the other hand, less amount of drug was loaded into GB-39 EVs^TMZ^ by indirect incubation, since it was 600 times less than the original exposure. Altogether, our results revealed that direct incubation is more efficient than the indirect method (Figure S4).

### 2.6. EPZ015666-Loaded GBM-Derived Small EVs Have a Similar effect on Cancer Cells

PRMT5 has recently emerged as a promising therapeutic target in GBM treatment. According to the UALCAN database, a web portal for gene expression and survival analysis in different types of tumors, PRMT5 was upregulated in most of the tumors (Figure S7A). This database, which uses data extracted from “The Cancer Genome Atlas” project, showed significant upregulation of PRMT5 in GBM samples (*p*-value 1.62 × 10^−12^) (Figure S7B) [39]. Moreover, its expression has been related to a high grade of malignancy in GBM, while its inhibition correlates with survival [40,41]. One of the PRMT5 inhibitors that have been successfully developed is EPZ015666 [14].

We wondered if small EVs could have a different target efficacy based on the loaded drugs. Along this line, different drugs with different mechanisms of action and chemical properties could be more suitable for small EVs drug delivery. To test the effect of EPZ015666 treatment on proliferation, an increasing concentration of the drug was used. A range of concentrations between 5 μM and 20 μM were applied to 3 cancer cell lines (Figure 6A,C). For this experiment, and to assess the effect in another GBM cell line, HGUE-GB-42 was used. Our analyses showed a nearly 50% decrease in proliferation in the GBM cell lines and a slightly higher reduction in RWP-1 (IC_50_) after EPZ015666 treatment (Figure 6A,C).

Small EVs were loaded by direct incubation in 15 μM EPZ015666 (GB-39 EVs^EPZ015666^). Similar to the previous experiments, HPLC analysis, followed by drug identification with a mass spectrometer, was performed (Figure S8). Our quantifications showed a 0.04 μM concentration in the small GB-39 EVs^EPZ015666^ by direct incubation. Therefore, those samples were loaded with a concentration that was 400 times less than the original 15 μM. Isolated small GB-39 EVs^EPZ015666^ were applied to the cells in serial dilutions ranging from 50% to 5% of the original stock. Consequently, the maximum concentration was 0.02 μM, corresponding to 50% small EVs, and the minimum was 0.002 μM when 5% of EVs^EPZ015666^ was applied. GB39-EVs^EPZ015666^ treatment resulted in a reduction in significant cancer cell proliferation. Confirming the tested idea, the higher decrease in proliferation took place in HGUE-GB-39, the cell line where the small EVs were obtained. An average of a 20.54% reduction in proliferation was seen in the RWP-1 cell line, regardless of the small EVs concentration (Figure 6B,C). These results indicate that even if this cell line does not have a specific affinity for this type of small EVs, the amount of EPZ015666 that reaches the cell is sufficient to induce an effect.

In accordance with our previous observations with GB-39 EVs^TMZ^, our HPLC data indicated that the amount of drug loaded into isolated GB39-EVs^EPZ015666^ (Figure S8) was much less than the concentration they were exposed to while being incubated by the direct method (Figure S8). Therefore, it confirms that direct incubation is the most efficient method to load GB39-EVs^EPZ015666^ (Figure S8).

## 3. Discussion

Glioblastoma patients with metastatic or relapsed disease have poor outcomes, despite intense and aggressive multimodal treatment strategies, including high-dose chemotherapy regimens. Current therapies are insufficient, with nearly universal recurrence, mostly due to the difficulty of delivering therapeutic agents to the brain. Since the BBB limits the entry of systematically administered drugs to the brain, efforts are underway to develop methods to deliver treatments behind the BBB, such as the implantation of a drug-eluting material into the brain tissue. Along this line, controlled release systems for direct delivery of chemotherapy to brain tumors were first approved by the FDA in 1996 [42,43]. The intensification of preclinical discovery efforts has led to the identification of novel drug delivery systems that are currently being investigated in clinical trials; however, the efficacy, and more importantly, the direct pharmacological targeting of tumor cells remains elusive [25]. Of note, while there are an increasing number of drug delivery systems that are currently being developed and tested in preclinical studies, only a few of them advance to clinical testing due to inherent limitations, such as toxicity to normal cells [44].

Recently, small EVs are being investigated and manipulated to be used as delivery vehicles for anticancer drugs and molecules [25]. Their nanosize, structure, and origin, together with the presence of several adhesion proteins on the surface that enhance fusion with recipient cells, make small EVs very good candidates to deliver therapeutics with increased efficiency and specificity to tumor cells [45,46]. Given the pressing need in GBM therapy for testing new drug delivery systems that are safe, exhibit preferential toxicity to tumor cells, and spare normal cells, we decided to investigate the possible application of small EVs in cancer, with a special focus on GBM. Hence, we performed a deep characterization of seven patient-derived GBM cell lines and their small EVs.

Small EVs have been tested as natural drug delivery vehicles for cancer treatment by loading them with a drug or molecule through several methods, including sonication, electroporation, or by transfecting the cells with the molecules of interest and then isolating the small EVs loaded with those molecules from the transfected cells [47,48,49]. Since methods such as electroporation and sonication are likely to produce structural or physiological changes in the small EVs, we loaded our small EVs by incubation methods with two drugs: TMZ and EPZ015666 [50,51].

The current standard-of-care treatment for GBM includes maximum safe surgical resection and radiotherapy with concomitant TMZ, followed by adjuvant TMZ. In most of the experiments performed, this drug was dissolved in DMSO, which is cytotoxic by itself [52]. Therefore, it is important to mention that in the experiments reported in this article, TMZ was dissolved in Milli-Q^TM^ water, as other authors have tested it [53]. Consequently, the effects on cell proliferation that we have described are solely relative to TMZ.

In the past few years, PRMT5 has been proposed as a possible therapeutic target for some cancers [12,13]. Not only is it dysregulated in several cancer types, but its expression has also been correlated with cancer patient survival. A detailed analysis of TCGA atlas samples with the internet tool cBioPortal (https://www.cbioportal.org/ accessed on 30 March 2022) shows that of the four GBM subtypes, PRMT5 is mostly upregulated in Classical GBM (Figure S7C) [54]. This is important, because the classical subtype shows a significant reduction in mortality with aggressive radiotherapy and chemotherapy [55]. In this article, we have shown for the first time the effect of the PRMT5 inhibitor, EPZ015666, on patient-derived GBM cell lines. More importantly, we have seen that EPZ015666 affects GBM cell proliferation more than TMZ already by direct application of the drug (Figures S1 and S2).

When we exposed GB-39 EVs to TMZ or EPZ015666, by direct or indirect incubation, we observed that both methods could load the small EVs. However, direct incubation with any of the drugs was more efficient in GBM patient-derived small EVs, as shown with the HPLC (Figures S4 and S8). Moreover, we observed that in the direct incubation method, a higher loading efficiency is obtained, depending on the hydrophobicity of the tested drugs, since they have a better interaction with the lipid bilayer of the small EVs membranes, as previously reported for other compounds (Figures S4 and S8) [56,57,58].

Interestingly, in vitro proliferation analysis led to the observation that drug-loaded small EVs of GBM cell lines affect GBM cells, regardless of the patient from whom they came. The alternative GBM cell line, which was very sensitive to both treatments being used, was also affected by GB-39 drug-loaded small EVs. Very low levels of those drugs already have an effect in that cell line, which can explain the effect of the loaded small EVs on its proliferation. In any case, we could rule out that the uptake of the GBM small EVs was specific since the effect on RWP-1 was not dosage-dependent.

GBM has an isocitrate dehydrogenase-wt phenotype (IDH wt), and it is considered as a grade 4 astrocytoma, according to WHO classification. On the other side, WHO 2021 classification indicates that IDH-wt LGG shows a molecular resemblance to GBM, and now it is also classified as such. Finally, there is another group that can also reach a grade 4, according to WHO classification, which is the Astrocytoma IDH-mutant that was previously considered as GBM, IDH-mutant [1]. The effect on the proliferation of this pancreatic cell line suggests that this delivery system could be used to target secondary metastasis [59]. Moreover, extracranial metastases of GBM are rare due to the short survival experienced by the patients, but they exist. These cases can benefit from the observation that GBM-derived small EVs can also affect other tumor cells; however, more analysis should be done to explore this possibility.

Several active efforts to re-evaluate existing or investigate new anti-GBM agents are ongoing, and our results expand the possibilities of investigating other combinatorial therapies. Another important aspect of GBM treatment includes the fact that chemotherapeutic regimens are largely non-selective and cytotoxic in nature, and despite all the efforts, almost all patients experience tumor progression, with nearly universal mortality and a median survival of less than 15 months, depending on several risk factors [60]. There is a need to optimize the balance between effectiveness and toxicity, and the use of small EVs may help to reduce the amount of drug given to a patient in order to reach the same effect. In this work, we measured the amount of drug loaded into the small EVs by HPLC to compare it with the direct application of those drugs. Specific studies are necessary to assess the therapeutic efficiency of the current drugs since, as we have shown, each drug has different loading properties. Importantly, this is the first work showing a detailed protocol to measure the EPZ015666 concentration by HPLC that can be applied to small EVs. HPLC analysis of loaded GBM small EVs has shown that very low amounts of EPZ015666 are more lethal than TMZ to cancer cells (Figures S1, S2, S4, and S8). Altogether, this work showed that small EVs can be loaded with an amount of at least 200 times less TMZ and an amount of 400 times less EPZ015666 and still be very efficient. This work opens a venue to work with GBM small EVs as drug delivery systems for GBM treatment.

## 4. Materials and Methods

### 4.1. Cell Culture

Isolation of primary human GBM cells was performed from surgical washes, as reported previously by Ventero and colleagues, and the cells were recently used to get some insight into GBM development [26,61]. The pancreatic adenocarcinoma (RWP-1) cell line was donated by Instituto Municipal de Investigaciones Médicas (IMIM, Barcelona, Spain), and it was recently used to understand not only the mechanism of cancer progression, but also to develop new delivery systems for cancer therapy [61,62]. Cells from the GBM cell lines HGUE-GB-16, HGUE-GB-18, HGUE-GB-37, HGUE-GB-39, HGUE-GB-40, HGUE-GB-42, and HGUE-GB-48 were cultured in Dulbecco’s Modified Eagle’s Medium: Nutrient Mixture F-12 (DMEM F-12) (Biowest, Riverside, MO, USA), whereas the RWP-1 cells were cultured in Dulbecco’s Modified Eagle’s Medium: High Glucose (DEMEM-HG) (Biowest, Riverside, MO, USA), and both were supplemented with 10% (*v*/*v*) of heat-inactivated fetal bovine serum (FBS) (Biowest, MO, USA) and 1% (*v*/*v*) of a penicillin/streptomycin mixture (Biowest, Riverside, MO, USA). The cells were incubated at 37 °C in a humidified 5% CO_2_ atmosphere.

### 4.2. Proliferation Assays

BCNU is a common treatment for GBM patients, together with radiotherapy. Therefore, we established a classification that includes not only TMZ and EPZ015666, but also BCNU and radiotherapy. We characterized the seven GBM cell lines according to their sensitivity and/or resistance to different chemotherapeutic drugs into three categories—Sensitive, Partially Sensitive, and Resistant—based on the IC_50_ calculated from the results obtained after cell exposure to drugs or X-ray radiation [26]. We performed a series of MTT experiments with EPZ015666 and TMZ, while previous data were analyzed to evaluate BCNU and radiotherapy resistance, according to this classification [26]. (Table 1, Figures S1 and S2).

Cells were seeded in 96-well standard plates (Sarstedt, Nümbrecht, Germany) with a density of 4000 cells/well and incubated at 37 °C and 5% CO_2_ for 24 h. After that, cells were treated with different and crescent concentrations of the chemotherapeutic drugs or loaded small EVs and incubated for 72 h under the same conditions. Then, 0.25 mg mL^−1^ of methylthiazolyldiphenyl-tetrazolium bromide (MTT) (Sigma-Aldrich, St. Louis, MO, USA) was added and incubated for 3 h, media were carefully removed, and 100 μL of dimethyl sulfoxide (DMSO) (Sigma-Aldrich, St. Louis, MO, USA) was added. The plate was vigorously shaken at room temperature for 20 min to dissolve the formazan crystals. Finally, the absorbance was measured on an Eon^TM^ Microplate Spectrophotometer (BioTeK^®^, Winooski, VT, USA) at 570 nm.

### 4.3. Small EVs Purification for Characterization

Confluent populations of the seven GBM cell lines were incubated in 75 cm^2^ cell culture flasks with DMEM F-12 conditioned media, and after 96 h without a medium change, small EVs were obtained by the differential ultracentrifugation method (Figure 2). The media samples were centrifuged at 2000× *g* for 20 min at 4 °C to eliminate cells and debris, followed by an initial ultracentrifugation using an Optima L-90K Ultracentrifuge (Beckman Coulter, Brea, CA, USA) with 70.1 Ti rotor at 25,000× *g* for 40 min at 4 °C to remove macrovesicles, and then the supernatant was filtered through 0.22 μm filters (Fisher Scientific, Pittsburgh, PA,USA). The resultant filtrate was centrifuged at 110,000× *g* for 90 min at 4 °C to pellet small EVs. Small EVs were washed in PBS at 110,000× *g* for 90 min at 4 °C, and the final pellet was resuspended in 200 μL of 1X PBS and then stored at −80 °C.

### 4.4. Western Blot

Protein samples were solubilized with a loading buffer (4×) (2-mercaptoethanol + NuPage; 1:5) and heated at 95 °C for 5 min for reducing conditions, and for non-reducing conditions, the loading buffer was used without 2-mercaptoethanol. Then, proteins were separated by SDS-PAGE using 10% acrylamide gels and transferred to a nitrocellulose membrane (Bio-Rad Laboratories Inc., Hercules, CA, USA). Membranes were incubated overnight at 4 °C with the primary antibodies anti-CD63 (mouse,1:1000, Clone Ts63, Invitrogen, Waltham, MA, USA), anti-ALIX (mouse,1:500, Clone 3A9, Invitrogen), anti-TSG101 (mouse,1:500, Clone 4A10, Invitrogen), anti-α-tubulin (mouse, 1:10,000, Clone DM1A, Invitrogen), and anti-HSP90-B1 (rabbit,1:4000, CUSABIO), followed by one hour of incubation at room temperature with ECL^TM^ Anti-mouse IgG and ECL^TM^ Anti-rabbit IgG, Horseradish Peroxidase linker (GE Healthcare, Chalfont St Giles, UK). The membranes were visualized with ECL^TM^ Prime Western blotting detection reagent (Amersham^TM^) in the ChemiDoc Bio-Rad instrument (Hercules, CA, USA).

### 4.5. Dynamic Light Scattering (DLS)

Small EVs samples were analyzed using a Zetasizer Nano ZS instrument (Malvern Panalytical, Worcestershire, UK). In order to characterize the size distribution, each sample was diluted in Milli-Q^TM^ water (1:10), and 1 mL was dispensed in a polystyrene disposable cuvette (Zen0040) and read seven times in Zeta sizer Software. The final image with the distribution curves was used in the Figures as illustrative of the medium size (Figure 3).

### 4.6. Field Emission Scanning Electron Microscope (FESEM)

Small EVs were vortex and fixed with paraformaldehyde 2%. The fixed small EVs were sonicated for 5 min and then diluted in serial dilutions with Milli-Q^TM^ water (1:10; 1:100; 1:1000, 1:10,000). A 50 μL sample droplet was deposited on a silicon wafer, and, once evaporated, the samples were observed using a Zeiss Sigma 300 VP Field Emission Scanning Electron Microscope (FESEM, Carl Zeiss, Oberkochen, Germany) without coating. The EVs morphology was analyzed at low voltages around 1 kV, while higher voltages were employed to differentiate the salts from the biological sample by using Energy Dispersive X-ray (EDX).

### 4.7. Small EVs Drug Loading

Small EVs, obtained as described in the small EVs purification for characterization section, were loaded by direct incubation. Small EVs were incubated for 2 h at 37 °C in a thermoblock with defined concentrations of a given chemotherapeutic drug (EPZ015666 15 μM or TMZ 5 mM). Then, they were washed with PBS 1X and ultracentrifuged at 110,000× *g* for 60 min at 4 °C to pellet loaded small EVs. Finally, they were filtered with a 0.22 μm membrane under sterile conditions and stored at −80 °C. For the indirect procedure, confluent populations of GBM cells were seeded, and after 24 h, they were treated with the drug of interest (EPZ015666 15 μM or TMZ 5 mM) for 72 h. Then, the small EVs were purified as described in the small EVs purification for characterization section.

### 4.8. Quantification of TMZ and EPZ015666 by HPLC

The amount of TMZ and EPZ015666 loaded into the small EVs was measured by the high-performance liquid chromatography (HPLC) method. Briefly, 200 μL of small EVs were placed in a Concentrator plus (Eppendorf, Germany) at 40 °C for 2 h to evaporate the solvent. Then, 100 μL volume of acetonitrile was added, and the mixture was vortexed, sonicated, and then centrifuged at 13,000 r.p.m (Centrifuge 5415 R, Eppendorf, Germany) for 10 min. After centrifugation, the supernatant was taken, transferred into HPLC vials, and injected into the UPLC-QtoF-MS/MS equipment with a high-resolution flight tube and quadrupole technology (Waters-Bruker). Detection of TMZ and EPZ015666 was optimized with a Waters I-Class with UV detection at 330 and 254 nm, respectively, and also a QToF-MS de Bruker Daltonics, model maXis impact Series, in the mode positive ionization by Electrospray (ESI) with column ACE Excel C18-Ar (50-3; 1.7 µm). The mobile phase used was (A) water with 0.1% (*v*/*v*) acetic acid and (B) methanol with 0.1% acetic acid. The gradient was set with the following conditions: 0 min, 95%A; 5.50 min, 60%A, 8.75 min, 5%A, with a flow rate of 0.3 mL/min and an injection volume of 5 μL.

### 4.9. Immunocytochemistry

HGUE-GB-39, HGUE-GB42, HGUE-GB-48, and RWP-1 cells were seeded on coverslips in 24-well plates (30,000 cells/well) and incubated at 37 °C and 5% CO_2_. After 24 h, cells were treated with crescent concentrations of the chemotherapeutic drugs or loaded small EVs and incubated for 72 h under the same conditions. Cells were fixed with paraformaldehyde 4% and blocked with FBS/PBS (1×) (50 μL/mL), followed by incubation with anti-ki67 (1:100, mouse; Invitrogen) primary antibody. After washing out the first antibody, cells were incubated with Alexa Fluor 568-labeled anti-mouse (1:500) secondary antibody (Invitrogen, Barcelona, Spain) and DAPI (4′,6-diamidino-2-phenylindole, Sigma) to stain the nucleus. Coverslips were mounted in Prolong™ Gold Antifade reagent (Invitrogen, OR, USA) and analyzed using a confocal microscope, LSM 900 (Carl Zeiss, Germany).

### 4.10. Statistical Analysis

Results are shown as the mean ± standard deviation (SD) of three independent experiments. In order to evaluate the normal distribution of the data, the Shapiro–Wilk statistical test was used, and the Student’s *t*-test or the Mann–Whitney U test was used to analyze the association between variables. Differences were considered to be statistically significant with a *p*-value of less than 0.05. To calculate the IC_50_ values, a nonlinear regression analysis was performed. Statistical analysis was performed with GraphPad Prism v7.0a software (GraphPad Software Inc., San Diego CA, USA).

## 5. Conclusions

In summary, we have demonstrated that TMZ and EPZ015666 inhibit the growth of GBM cells in vitro by less exposure to the drug when this is loaded in small EVs. In this work, EPZ015666 content was measured by HPLC for the first time in EVs, and our results showed that direct incubation was a better loading method for small EVs. We challenged the hypothesis that small EVs tend to fuse with their own mother cell, and, accordingly, we observed a dose-dependent proliferation effect in GBM cell lines, but not in the pancreatic cancer cell line. The fact that other cancer cells can be affected, although to a lesser extent, by drug-loaded GBM EVs entails their possible application to secondary metastasis. Collectively, these results suggest a potential use of GBM-derived small EVs in drug delivery to obtain the maximum therapeutic effect with minimal toxicity in the treatment of GBM patients.

## Figures and Tables

**Table 1:** **Sensitivity and/or resistance classification of GBM lines to treatments.** GBM cell lines classification according to their sensitivity and/or resistance to EPZ015666, TMZ, Carmustine (BCNU), and radiotherapy treatments. Cells were treated with different concentrations of the chemotherapeutic agents EPZ015666 and TMZ. Classification of BCNU and radiotherapy data was performed based on data previously reported by our research group [26]. Classification of those treatments is based on the IC_50_ of each one. The resistant cell lines of each treatment are highlighted in red and the sensitive ones are highlighted in light green. GBM, glioblastoma; TMZ, Temozolomide.

	Treatment	EPZ015666	TMZ	BCNU	Radiotherapy
Cell Line					
**HGUE-GB-16**		Partially sensitive	Sensitive	Resistant	Sensitive
**HGUE-GB-18**		Partially sensitive	Sensitive	Partially sensitive	Partially sensitive
**HGUE-GB-37**		Partially sensitive	Sensitive	Partially sensitive	Sensitive
**HGUE-GB-39**		Sensitive	Sensitive	Partially sensitive	Resistant
**HGUE-GB-40**		Partially sensitive	Sensitive	Partially sensitive	Partially sensitive
**HGUE-GB-42**		Sensitive	Sensitive	Sensitive	Sensitive
**HGUE-GB-48**		Partially sensitive	Sensitive	Partially sensitive	Partially sensitive

## Data Availability

The data that support the findings of this study are available from the corresponding author, C.d.J.R., upon reasonable request.

## Supplementary Materials

The following supporting information can be downloaded at: https://www.mdpi.com/article/10.3390/ijms24065910/s1.
