# Supplementary material for: Glioblastoma-Derived Small Extracellular Vesicles: Nanoparticles for Glioma Treatment
Source: Int J Mol Sci. 2023 Mar 21;24(6):5910. doi: 10.3390/ijms24065910 (PMC10054028; doi:10.3390/ijms24065910)
Supplement: Supplementary file 1 [file ijms-24-05910-s001.zip › ijms-2303835-supplementary.pdf]

Supplementary Figures

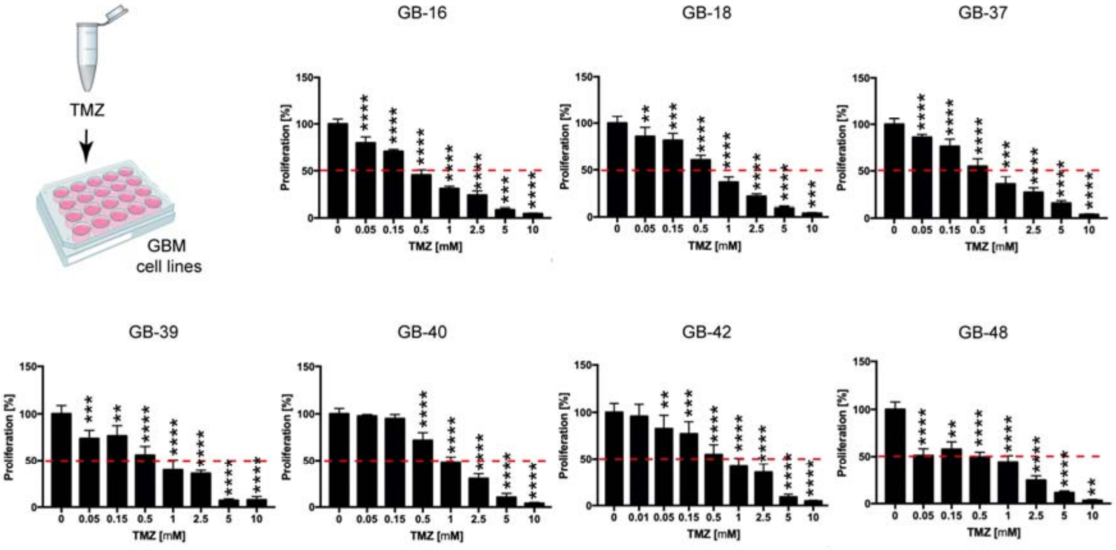

GBM cell lines	GB-16	GB-18	GB-37	GB-39	GB-40	GB-42	GB-48
IC_{50}	0.92	1.47	1.44	1.08	1.96	1.13	0.22

Figure S1. TMZ proliferation studies of GBM cell lines. Cancer cells were treated with increasing concentrations of TMZ and their proliferation was measured. Red line indicates IC_{50}. Asterisks indicate the statistical significance of the results (**p<0.01, ***p<0.001, ****p<0.0001). GBM, Glioblastoma; TMZ, Temozolomide.

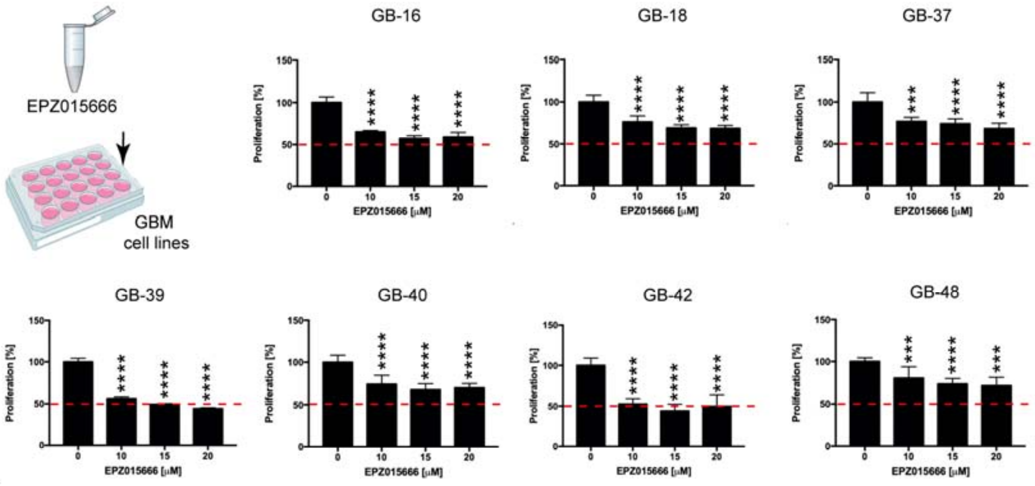

Figure S2. EPZ015666 proliferation studies of GBM cell lines. Cancer cells were treated with increasing concentrations of EPZ015666 and their proliferation was measured. Red line indicates IC_{50}. Asterisks indicate the statistical significance of the results (***$p<0.001$, ****$p<0.0001$). GBM, Glioblastoma.

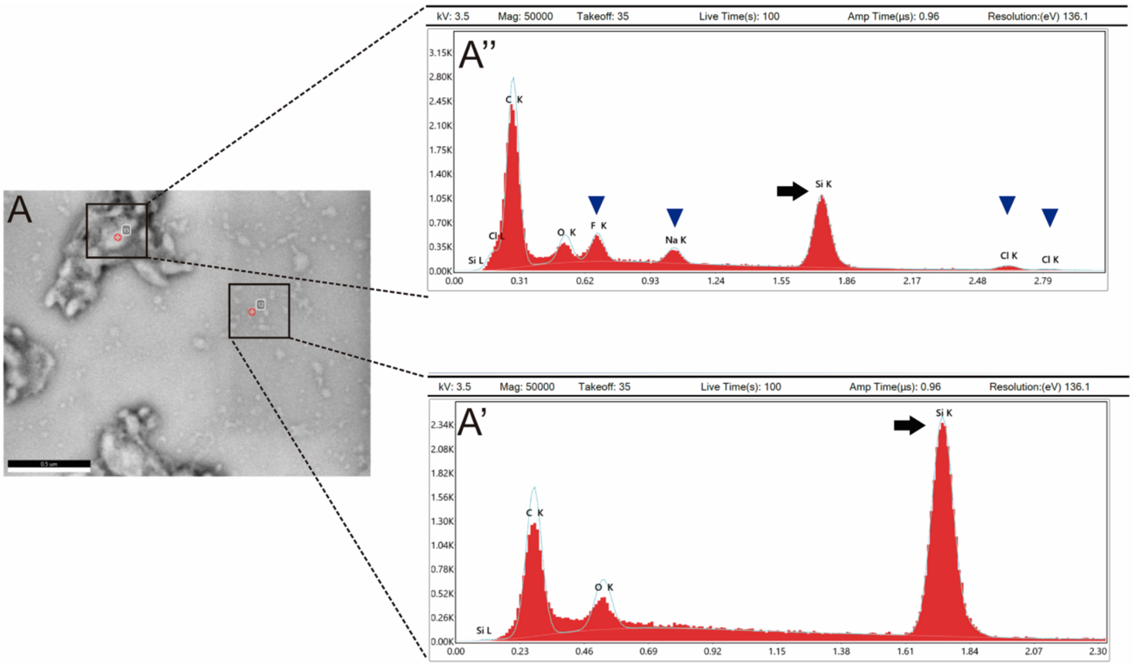

Figure S3. EDX analysis of small EVs sample HGUE-GB-48. (A) Energy dispersive X-ray (EDX) analysis was performed with the X-ray detector in a FESEM microscope on one of the EVs samples. The studied samples were a mixture of biological samples and salts. Black boxes in the pictures indicate the location of the EVs analysis (A') and salts (A''). The spectrum of each of the areas was done at 3.5 kV. Black arrows indicated the silica peak (Si) corresponding to the substrate. Blue arrowheads point at different inorganic elements present in salt crystals but not in the EVs. Scale bar, 0.5 µm. EVs, extracellular vesicles; FESEM, Field Emission Scanning Electron Microscope.

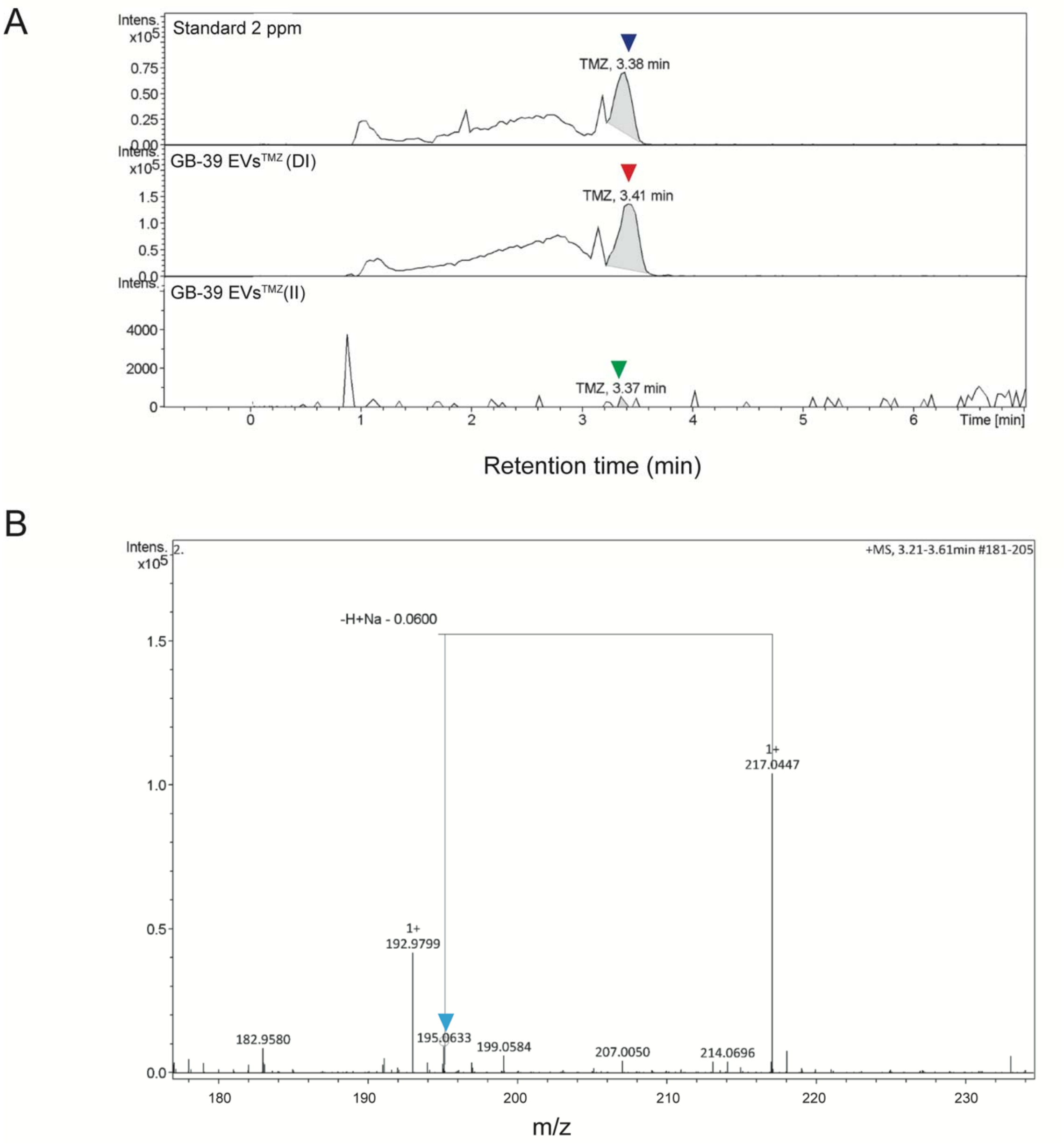

Figure S4. **HPLC-MS analysis of small EVs sample GB-39 EVs^{TMZ}.** (A) The amount of TMZ incorporated into small EVs was quantified by HPLC. The dark blue arrow indicates the standard TMZ (2 ppm) at 3.38 min retention time. The red arrow indicates the GB-39 EVs^{TMZ} sample (Direct Incubation) (5.028 µg/mL) at a retention time of 3.41 min. (B) Samples were confirmed with a mass spectrometer, as shown by the light blue arrow indicating the presence of TMZ (195.06 m/z). TMZ, Temozolomide; EVs, extracellular vesicles; m/z, mass-to-charge ratio.

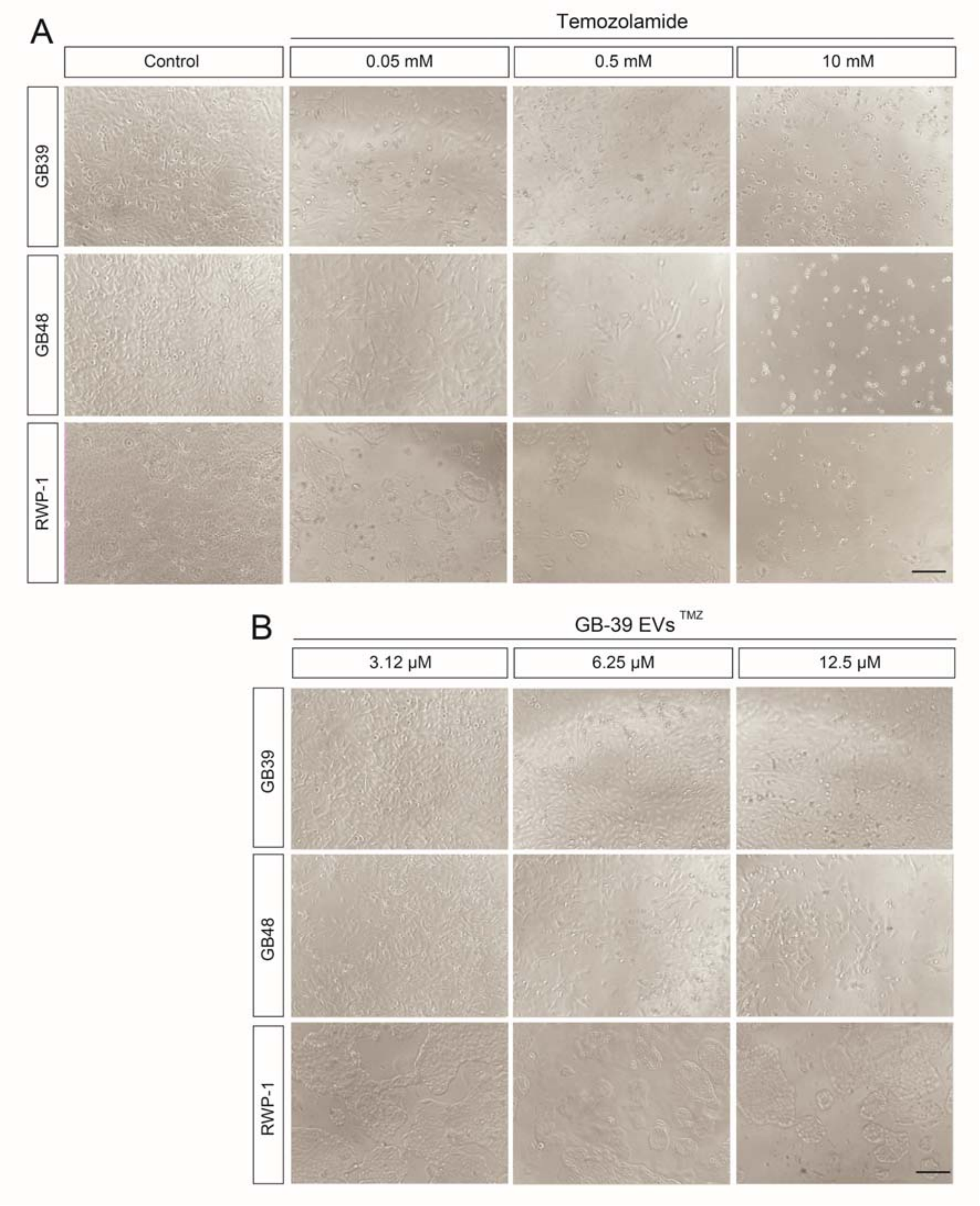

Figure S5. Proliferation in cancer cells after TMZ treatment. (A) Visualization of the decrease in proliferation of several cancer cell lines after exposure to increasing concentrations of TMZ; (B) Proliferation effect on the cancer cell lines of small EVs derived from GB-39 loaded with increasing concentrations of TMZ. In boxes are indicated the amount of TMZ loaded into the small EVs. TMZ, Temozolomide; EVs, extracellular vesicles. Scale bar A, B: 250 μm

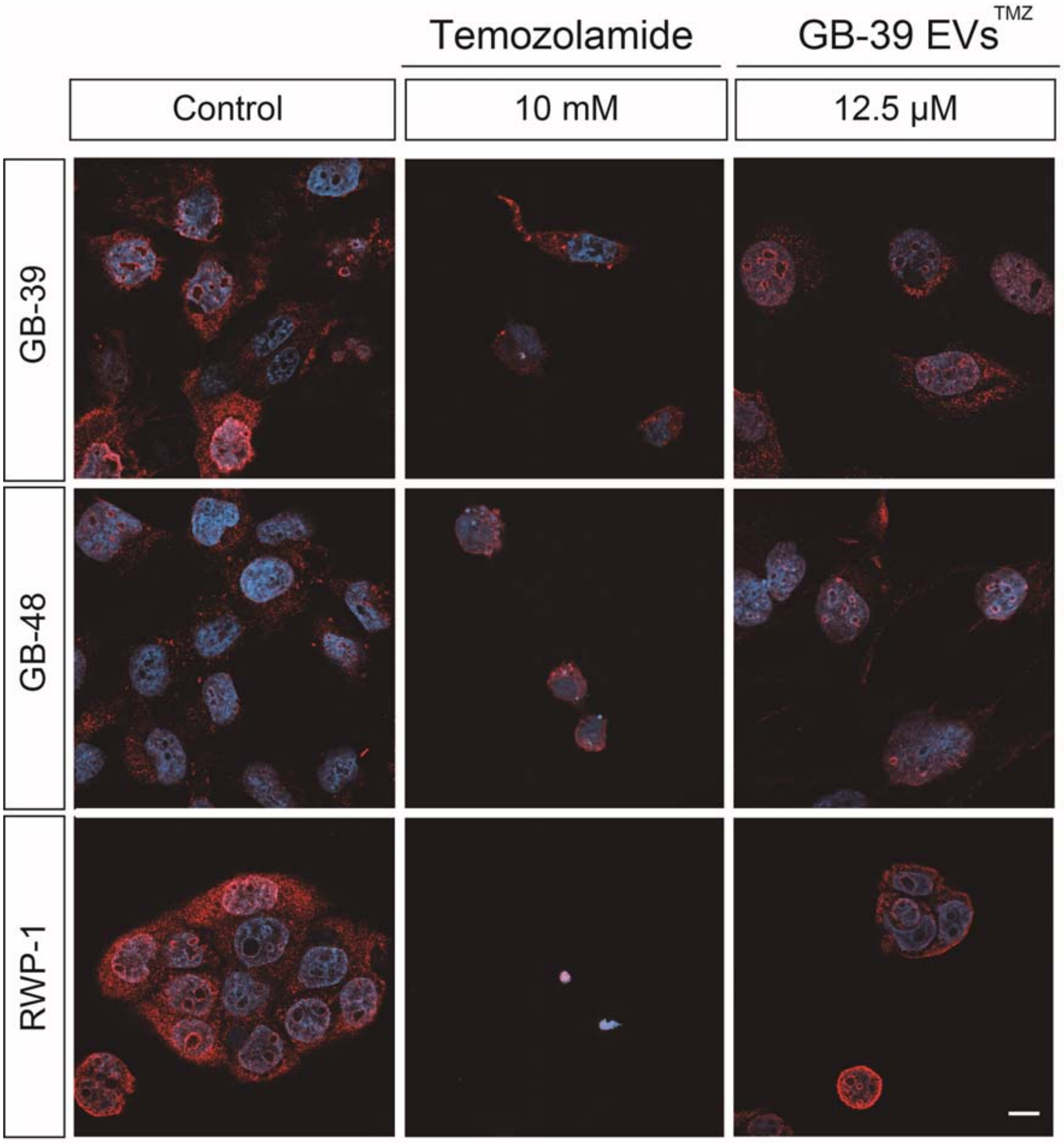

Figure S6. Visualization of proliferation in cancer cells after TMZ treatment. Confocal images of cancer cell lines after exposure to increasing concentrations of TMZ. Nuclei are visualized with DAPI (blue) and Ki67 (red) has been used as a marker for proliferation. Cells were stained after direct exposure to TMZ (A) or GB-39 EVs^{TMZ} (B). The boxes indicate the amount of TMZ loaded into the small EVs. Scale bar 10 μm. TMZ, Temozolomide; EVs, extracellular vesicles.

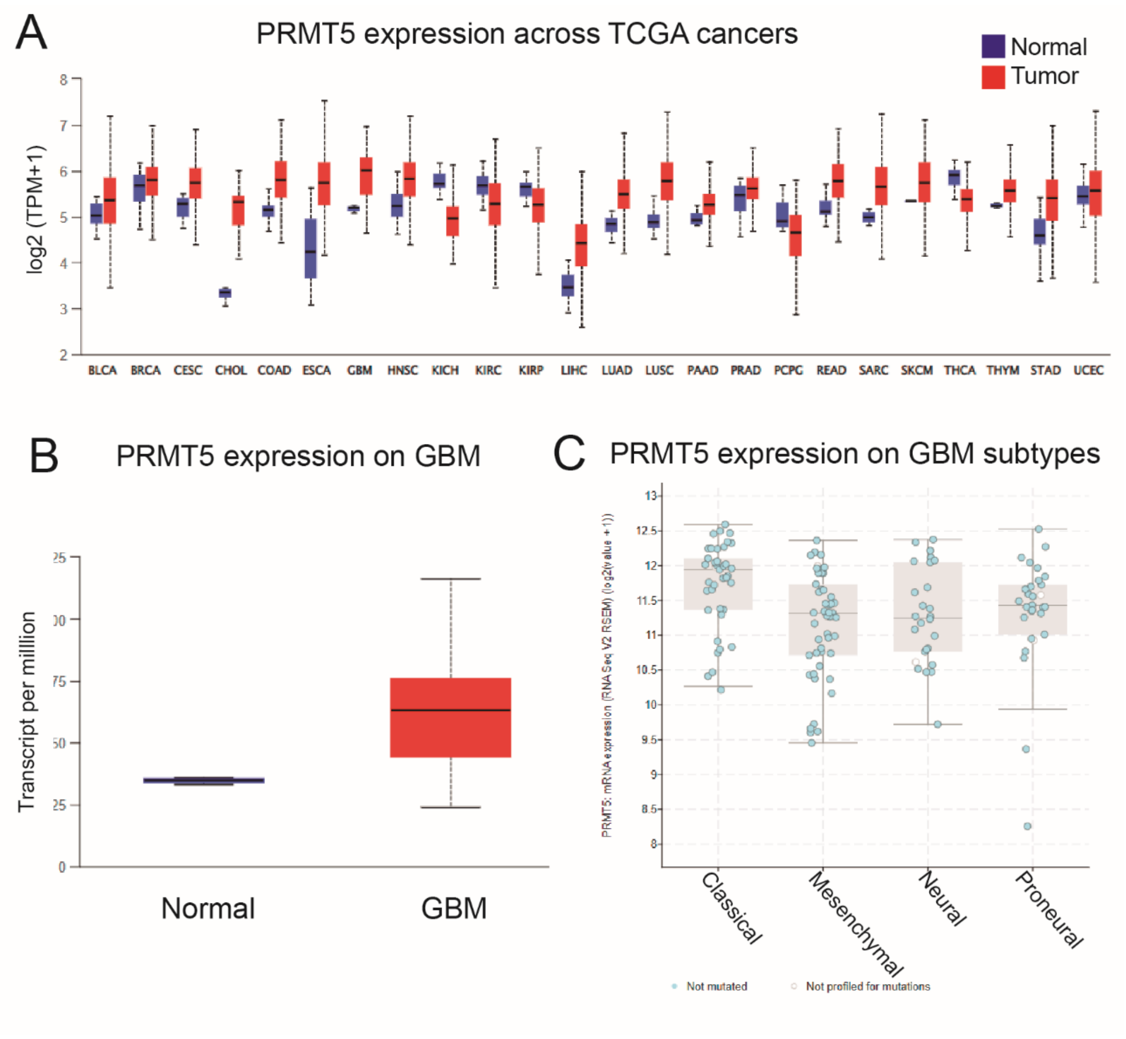

Figure S7. PRMT5 expression in cancer. (A) Expression analysis in Ualcan of PRMT5 across TCGA cancers. (B) Expression of PRMT5 in normal (n=5) and GBM samples (n=156) performed with Ualcan. (C) Expression analysis performed with cBioPortal comparing the expression of PRMT5 in the four subtypes of GBM. Blue boxes, normal; red boxes, tumor; blue circle, not mutated; white circle, not profiled for mutations; COAD, Adrenocortical carcinoma; BLCA, Bladder urothelial carcinoma; BRCA, Breast invasive carcinoma; CESC, Cervical squamous cell carcinoma; CHOL, Cholangiocarcinoma; COAD, Colon adenocarcinoma; ESCA, Esophageal carcinoma; GBM, Glioblastoma multiforme; HNSC, Head and Neck squamous cell carcinoma; KICH, Kidney Chromophobe; KIRC, Kidney renal clear cell carcinoma; KIRP, Kidney renal papillary cell carcinoma; LIHC, Liver hepatocellular carcinoma; LUAD, Lung adenocarcinoma; LUSC, Lung squamous cell carcinoma; PAAD, Pancreatic adenocarcinoma; PRAD, Pheochromocytoma, and Paraganglioma; PRAD,

Prostate adenocarcinoma; READ, Rectum adenocarcinoma; SARC, Sarcoma; SKCM, Skin Cutaneous Melanoma; THCA, Testicular Germ Cell Tumors; THYM, Thymoma, and Thyroid carcinoma; UCEC, Uterine Carcinosarcoma.

A

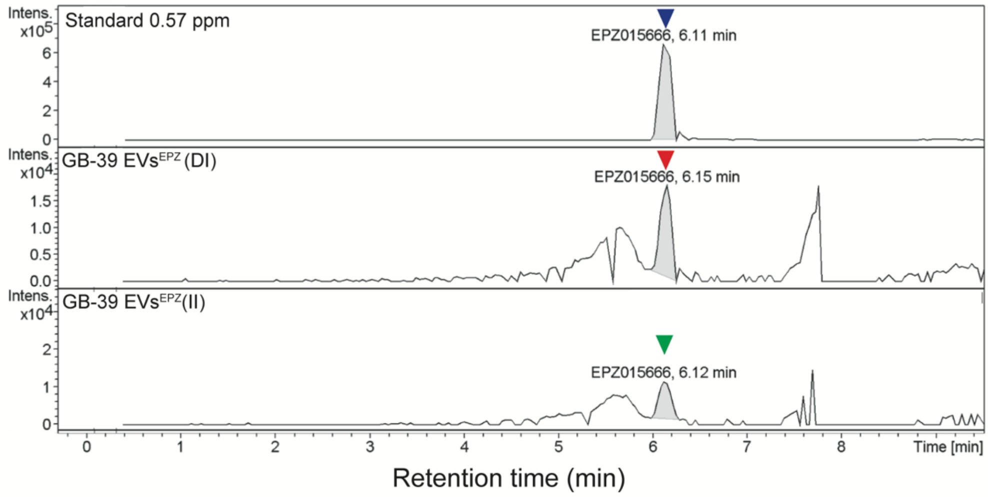

B

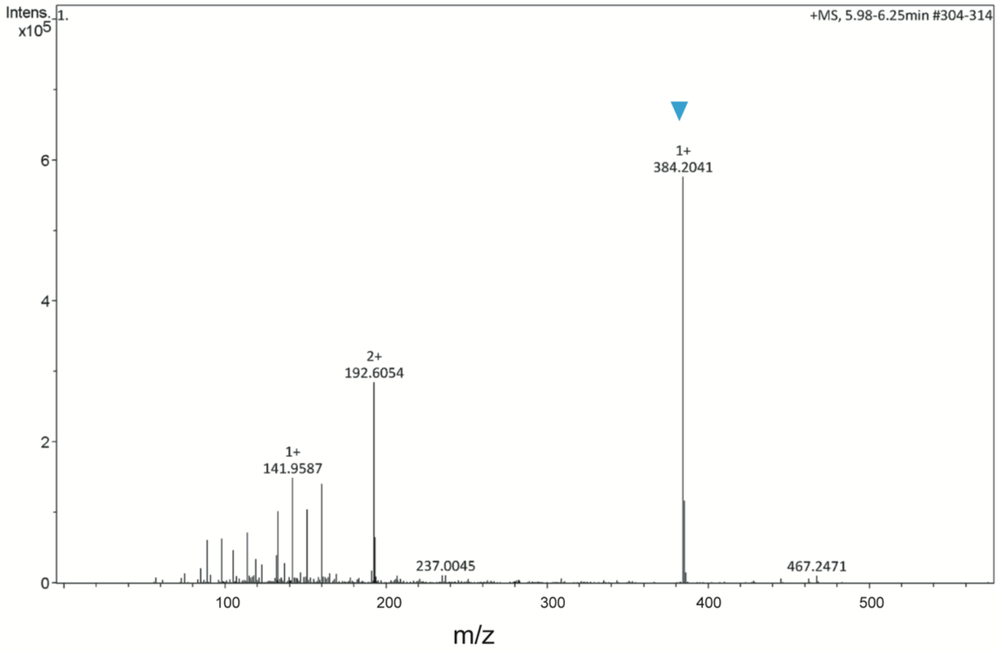

Figure S8. **HPLC-MS analysis of small EVs sample GB-39 EVs^{EPZ015666}.** (A) The amount of EPZ015666 incorporated into small EVs was quantified by HPLC. The dark blue arrow indicates the standard EPZ015666 (0.57 ppm) at a 6.11 min retention time. Red arrow indicates the GB-39 EVs^{EPZ015666} sample (Direct Incubation) (0.01058 µg/mL) at 6.15 min retention time. Green arrow points to the sample GB-39 EVs^{EPZ015666} (Indirect incubation) (0.00547 µg/mL) at 6.12 min retention time. (B) Samples were confirmed with a mass spectrometer, as shown by the light blue arrow indicating the presence of EPZ015666 (384.20 m/z). EVs, extracellular vesicles; m/z, mass-to-charge ratio.

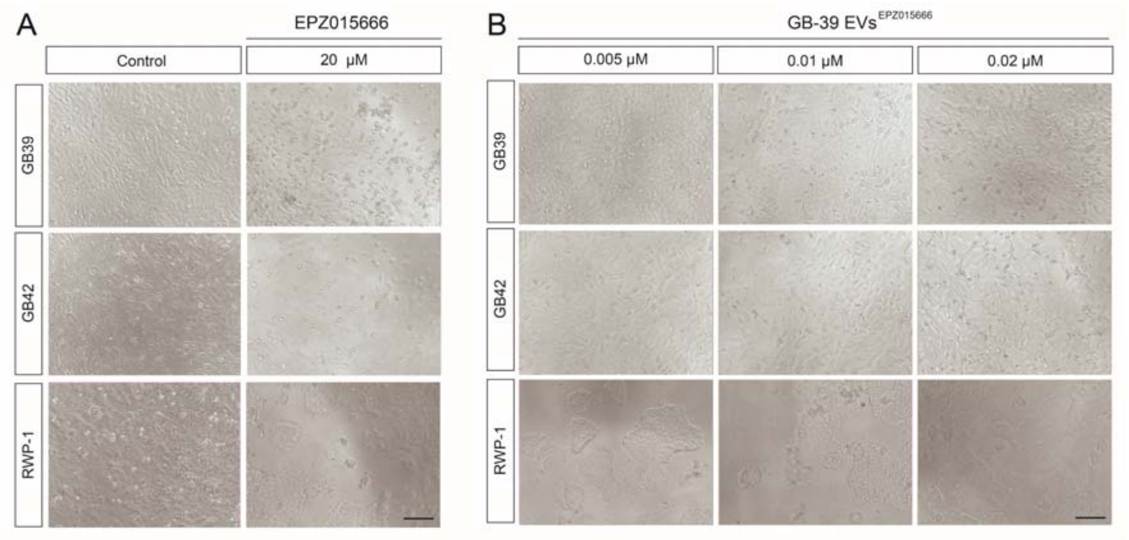

Figure S9. Proliferation in cancer cells after EPZ015666 treatment. (A) Visualization of the decrease in proliferation of several cancer cell lines after exposure to the higher concentration of EPZ015666; (B) Proliferation effect on the cancer cell lines of small EVs derived from GB-39 loaded with increasing concentrations of EPZ015666. In boxes are indicated the amount of EPZ015666 loaded into the small EVs. EVs, extracellular vesicles. Scale bar A, B: 250 μm

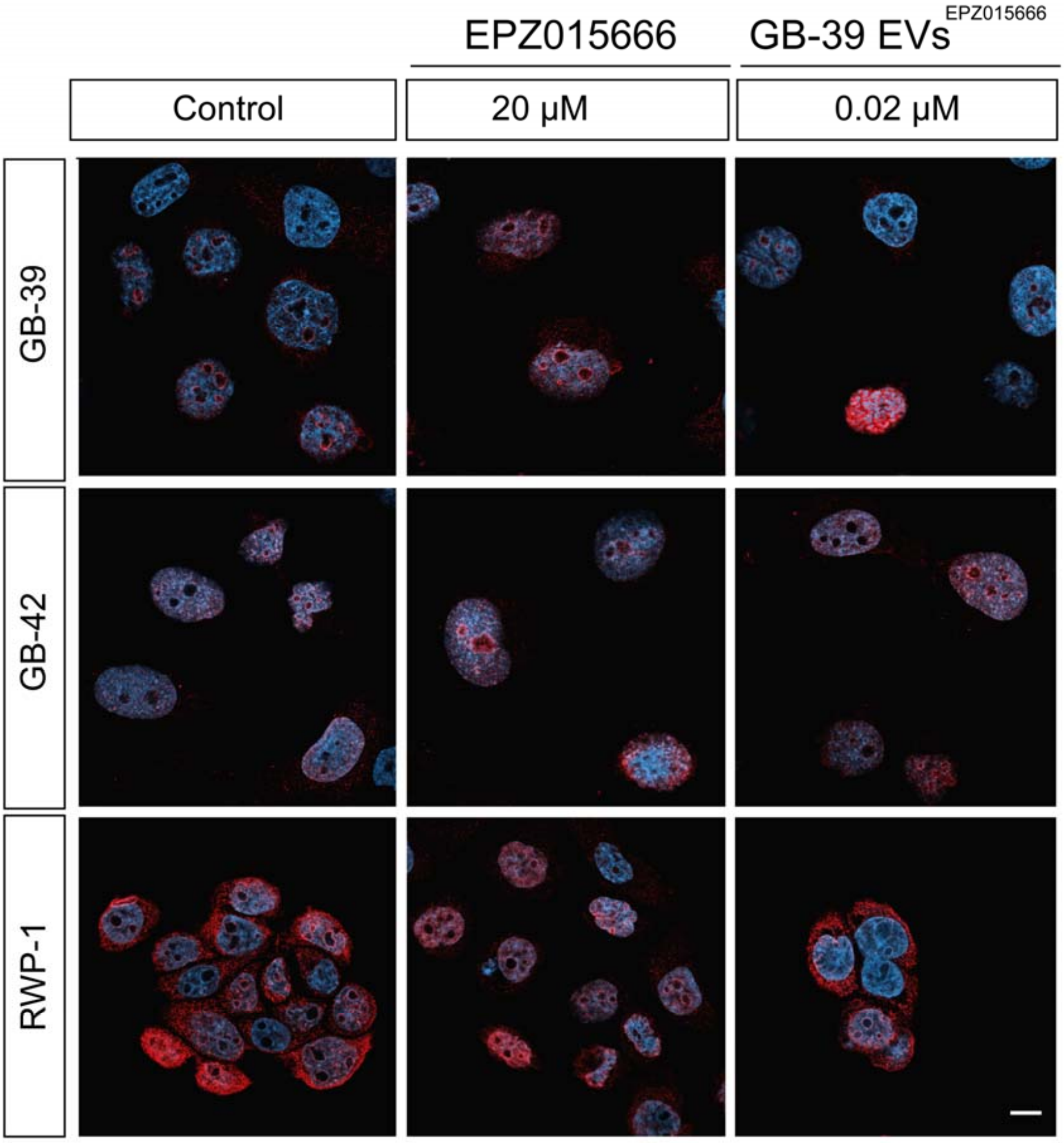

Figure S10. Visualization of proliferation in cancer cells after EPZ015666 treatment. Confocal images of cancer cell lines after exposure to increasing concentrations of EPZ015666. Nuclei are visualized with DAPI (blue) and Ki67 (red) has been used as a marker for proliferation. Cells were stained after direct exposure to EPZ015666 (A) or GB-39 EVs ^{EPZ015666} (B). In boxes are indicated the amount of EPZ015666 loaded into the small EVs. Scale bar 10 μm. EVs, extracellular vesicles.
